# The Utility of PET/CT for the Diagnosis of Periosteal Chondrosarcoma in a Patient With Maffucci’s Syndrome

**DOI:** 10.7759/cureus.46552

**Published:** 2023-10-05

**Authors:** Atishay Jain, James Cassuto, Efrosyni Sfakianaki, Russ A Kuker, Hamza Alizai, Sohaib Mohiuddin

**Affiliations:** 1 Research and Innovation, Green Medical Network Group, Bridgewater, USA; 2 Radiology and Nuclear Medicine, Overlook Medical Center/Atlantic Medical Group, Summit, USA; 3 Nuclear Medicine, Jackson Memorial Hospital, Miami, USA; 4 Radiology and Nuclear Medicine, Jackson Memorial Hospital, Miami, USA; 5 Radiology, Children’s Hospital of Philadelphia/University of Pennsylvania, Philadelphia, USA; 6 Radiology and Nuclear Medicine, Lehigh Valley Health Network, Allentown, USA

**Keywords:** 18fdg pet, maffucci’s syndrome, fdg pet/ct, multiple enchondromatosis, below-the-knee amputation, periosteal chondrosarcoma, enchondroma

## Abstract

Maffucci’s syndrome is a rare congenital nonhereditary syndrome with less than 300 cases having been reported in the United States. It is characterized by multiple enchondromas, hemangiomas, and rarely lymphangiomas. Enchondromas may undergo malignant transformation to chondrosarcomas. Surveillance plays a vital role in detecting early malignant transformation. Fluorodeoxyglucose (FDG) PET/CT, although falling out of favor, may be utilized as an imaging modality by physicians to determine such transformation, allowing for timely management and intervention. In this report, we share our experience with such a case.

## Introduction

Angelo Maffucci first described Maffucci’s syndrome in 1881. In 2002, the World Health Organization classified Maffucci’s syndrome as a subtype of enchondromatosis [1]. Maffucci’s syndrome is a rare congenital nonhereditary mesodermal dysplasia that affects males and females at an equal rate [1]. Patients are normal at birth. The syndrome develops by the age of six in 45% of cases, and symptoms develop before puberty in 78% of cases. It is characterized by the occurrence of multiple enchondromas, hemangiomas, and, in rare instances, lymphangiomas, all of which may progress or undergo malignant transformation, although its likelihood is low [2]. Various imaging modalities such as X-ray and MRI are used for monitoring malignant transformation, and the utility of PET/CT imaging in the clinical workup is discussed in this case report.

## Case presentation

Clinical information

**Figure 1:**
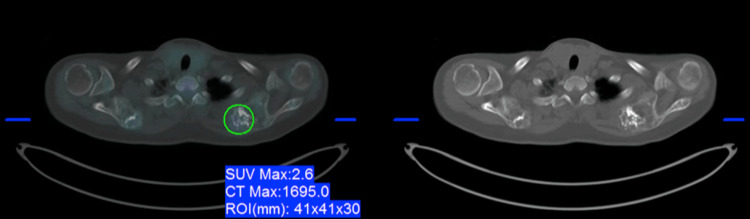
CT and fused PET/CT axial images of the superior thoracic region showing an expansile cartilaginous lesion in the left scapula demonstrating low-grade uptake (SUV: 2.6) CT: computed tomography, PET: positron emission tomography, SUV: standardized uptake value

**Figure 2:**
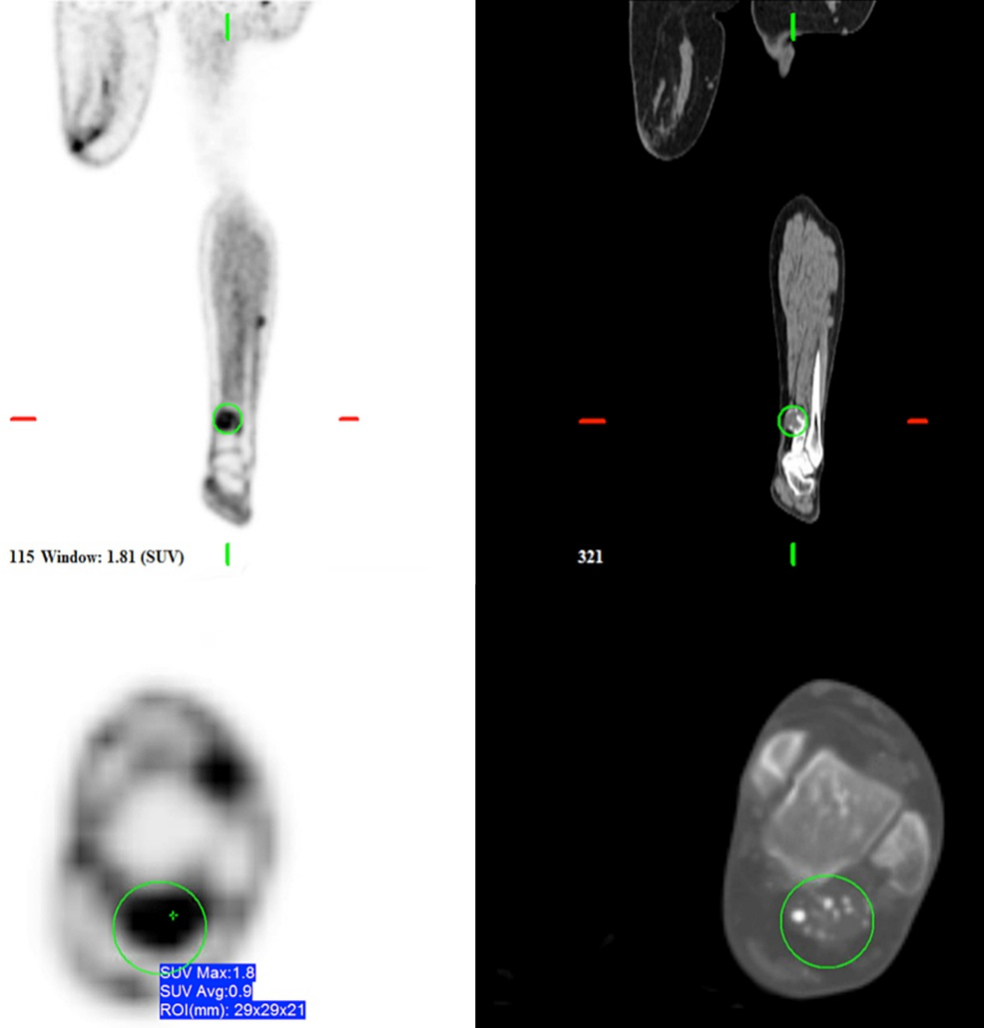
PET and CT coronal and axial images of the bilateral lower extremities demonstrating low-grade uptake (SUV: 1.8) lesions in the left lower leg CT: computed tomography, PET: positron emission tomography, SUV: standardized uptake value

A 38-year-old female with a known history of Maffucci’s syndrome presented with pain in her right distal lower extremity in June 2016. An MRI of the right ankle revealed a 1.9 cm mass along the right distal tibia extending laterally to the fibula. It was biopsied and reported to be a chondroma. One month later, a wide local excision of the same lesion was performed, and surgical pathology reported it to be juxtacortical myxoid chondrosarcoma (grade 2-3) with no vascular invasion and negative margins. An *IDH1* mutation was also detected. A few months later, the patient underwent right below-the-knee amputation for the right tibial chondrosarcoma, signifying the high-risk nature of the patient’s malignancy. Because of concern for further malignant transformation of enchondromas, the patient was closely monitored. A skeletal survey of radiographs showed multiple enchondromas. One lesion on the left scapula was evaluated with MRI, and a biopsy showed enchondroma. Additional lesions were detected on MRI, and a decision was made to do a fluorodeoxyglucose (FDG) PET/CT scan to help with the next steps given the potential for malignant transformation that had resulted in the amputation of the right leg. Lesional FDG uptake in Maffucci’s syndrome is reported to be variable but lacks cumulative evidence regarding FDG avidity. In our patient, the PET/CT findings helped with decision-making since many of the PET/CT SUV showed mild uptake and did not warrant surgical intervention. For example, the lesions in the left scapula (Figure 1) showed a heterogeneous expansile chondroid matrix with mild FDG uptake (SUV: 2.6). A more suspicious lesion in the left lower extremity tibial lesion (Figure 2) demonstrated a heterogeneous osseous lesion extending into the soft tissues with chondroid matrix appearance and lower mild FDG uptake (SUV: 1.8). Given the helpful information from the PET/CT that confirmed that the new left-sided lesions were of lower risk, the patient avoided having to undergo any additional surgical intervention. Close clinical follow-up was recommended due to the potential for malignant transformation.

This patient also had a history of nail-patella syndrome, a rare autosomal dominant disease of the connective tissue, classically producing absent/hypoplastic nails from birth and defects of the patella. There is a reported association between nail-patella syndrome and colon cancer; however, there is no association with bone tumors. This syndrome did not have any bearing on this case report.

## Discussion

Enchondromas are benign cartilaginous tumors growing in the medulla of bones, predominantly long bones. They most commonly occur in the hands, feet, femur, tibia, and fibula. Their distribution is asymmetric. They may result in bone disfigurations and limb shortening and bear the risk of pathological fractures in metaphyses and diaphyses. Soft tissue hemangiomas are vascular lesions and do not necessarily occur near bones or enchondromas. They have nodularity and firmness that many times contain calcium stones (phleboliths). They most commonly occur on the distal extremities but can appear anywhere in the body such as the leptomeninges, eyes, pharynx, tongue, oral mucosa, trachea, and intestine. Maffucci’s syndrome is also associated with a higher risk for malignancy of the ovaries, nervous system, pancreas, and liver [1-5].

Maffucci’s syndrome and its rare malignant transformation have rarely been reported with PET/CT findings. For instance, one case report of a 45-year-old male with multiple enchondromas and soft tissue hemangiomas showed low-grade FDG uptake (SUV: 1.8) within the enchondromas, and no FDG uptake in the soft tissue hemangiomas confirmed there was no malignant transformation [6]. A second case report of a 42-year-old male that demonstrates PET/CT findings of high-grade FDG uptake (SUV: 9.0) in a histologically proven aggressive myxoid chondrosarcoma was different from our case that had low-grade FDG uptake [7].

Diagnosis

The diagnosis can be classified as radiological, histological, and clinical.

Radiology

Enchondromas can be radiolucent or mineralized and can be intramedullary or periosteal in location. Phleboliths (soft tissue calcification) in hemangiomas can be visualized in radiographs. FDG PET appearance can be variable ranging from mild to moderate, to high uptake, and the average age for malignant transformation of enchondroma to chondrosarcoma in Maffucci’s syndrome patients is approximately in the fourth decade [6,7].

Histology

Enchondroma is a chondroid tumor with a slow growth pattern, mild biologic course, and regular lobulation pattern. The fibrous capsule has little blood vessels and low cellularity. It has hyaline cartilage nodules surrounded by lamellar bone [8]. Hemangiomas can be divided into two groups: capillary hemangiomas (narrow, thin-walled capillaries and thin epithelium separated by connective tissue) and cavernous hemangiomas (sharply defined and having deeper structures more often than capillary subtype). Another specific subtype called spindle cell hemangioma (features such as cavernous hemangioma combined with Kaposi sarcoma-like features) can be seen [9].

Molecular Diagnostics

In enchondromas and chondrosarcomas, mutations of the *PTHR1* gene were reported to be a candidate gene; however, subsequent studies could not confirm it. Somatic heterozygous mutations in *IDH1* or *IDH2* were reported in enchondromas and spindle cell hemangiomas [10]. Mutations are absent in DNA isolated from the blood, muscle, or saliva of the subjects. Therefore, these mutations are believed to be somatic.

Malignant transformation

The risk of malignancy in Maffucci’s patients shows an overall incidence anywhere between 23% and 100% as reported in the literature [7,11]. The majority of malignancies are of mesenchymal origin and include secondary chondrosarcoma and angiosarcoma. Most malignant transformations occur in the scapula and pelvic location rather than tubular bones [12].

Treatment

Regular physical examinations to evaluate changes in skin and bone lesions that may suggest malignancy are recommended. Although benign cases should be followed up radiographically, enchondromas may be biopsied if symptomatic [13]. Surgery may be done in cases of borderline or low-grade chondrosarcoma. Our patient had biopsy-proven chondrosarcoma of the tibia that resulted in a right below-the-knee amputation. Orthopedic specialists are required many times to address skeletal irregularities that may need surgical intervention [14]. Hemangiomas can be treated with laser therapy, sclerotherapy, or surgery to reduce the size of the lesions. Injecting a sclerosing agent will shrink and harden the area; however, operative removal is often needed [14].

## Conclusions

Maffucci’s syndrome is a congenital nonhereditary syndrome with a potential for malignant transformation, making it an important condition to monitor. This case highlights the importance of using FDG PET/CT as an imaging modality to differentiate low-risk versus high-risk patients who can undergo malignant transformation. To be implemented effectively, more evidence needs to be collected in order to determine SUV thresholds as low-grade chondrosarcomas can be difficult to differentiate from benign enchondromas that can show low-level FDG uptake.

Although high-grade chondrosarcomas correspond to high-level FDG uptake, the clinical scenarios of Maffucci’s syndrome patients demonstrate the utility of PET/CT for managing such complex cases. Unlike Maffucci’s syndrome, other more prevalent congenital enchondromatosis such as Ollier disease can be diagnosed by physical examination due to the lack of soft tissue hemangiomas, only further reinforcing the need for more evidence of FDG PET/CT studies to be completed on Maffucci’s syndrome patients.
